# Draft genome sequence of a clinical strain of *Kluyvera sichuanensis* isolated from human biliary drainage fluid

**DOI:** 10.1128/mra.01513-25

**Published:** 2026-04-20

**Authors:** Lina Liu, Yu Feng, Hongxia Wen, Zhiyong Zong

**Affiliations:** 1 Laboratory of Pathogen Research, West China Hospital, Sichuan University 12530, Chengdu, China; 2 Division of Infectious Diseases, State Key Laboratory of Biotherapy 429364, Chengdu, China; 3 Center of Infectious Diseases, West China Hospital, Sichuan University 12530, Chengdu, China; University of Pittsburgh School of Medicine, Pittsburgh, Pennsylvania, USA

**Keywords:** *bla*_CTX-M_, *Kluyvera*, *Kluyvera sichuanensis*

## Abstract

The genus *Kluyvera* (*Enterobacteriaceae*) comprises gram-negative, motile, non-spore-forming rods. *Kluyvera sichuanensis* strain 142937 was isolated in this study from biliary drainage fluid. The draft genome sequence of this clinical strain was determined to be approximately 5.09 million bases in length and harbors the resistance genes *bla*_CTX-M_, *fosA*, and *tet*(34).

## ANNOUNCEMENT

*Kluyvera* (*Enterobacteriaceae*) comprises five validly published species: *Kluyvera ascorbata*, *Kluyvera cryocrescens*, *Kluyvera georgiana*, *Kluyvera intermedia*, and *Kluyvera sichuanensis* (1–4). *Kluyvera sichuanensis* was identified in 2020 (1). Due to the scarcity of data about *K. sichuanensis*, particularly of clinical origin, we report the genome sequence of a strain isolated from a patient with biliary tract disease.

Strain 142937 was recovered from biliary drainage of a choledocholithiasis patient as part of routine care for the suspected biliary tract infection at our hospital in 2025. The sample was inoculated onto blood agar and cultured aerobically at 37°C for 24 h. A single colony was cultured in LB broth at 37°C for 18 h with aeration. DNA was extracted using the QIAamp DNA Blood Mini Kit (Qiagen, Hilden, Germany). A library was prepared with NEBNext Ultra II Kit (New England Biolabs, Ipswich, MA, USA), and 150 bp paired-end sequencing was performed on Illumina NovaSeq 6000 (Illumina, San Diego, CA, USA). Reads were trimmed by 10 bp from both ends using Cutadapt v5.2 (5). Adapter removal and quality filtering used BBMap v39.33 (https://jgi.doe.gov/data-and-tools/software-tools/bbtools), with a minimum quality of Q15 and length of 50 bp. Processed reads were assembled using SPAdes v4.2.0 (6). The genome assembly was deposited in NCBI and annotated with PGAP.

Species identification was confirmed by average nucleotide identity (ANI) using FastANI v1.34 (7). Plasmid replicons and resistance genes were identified using PlasmidFinder (https://genomicepidemiology.org) and ABRicate v1.0.1 (ResFinder database), respectively. Sequencing generated 8,903,790 reads (261× coverage), assembled into a 5,099,039 bp draft genome (46 contigs, N50 = 468,759 bp, 55% GC). Plasmid replicon IncFIA was identified. Strain 142937 is *K. sichuanensis*, with 97.39% ANI to GDMCC1.1872^T^ (GenBank accession number: JABBJF000000000). Strain 142937 had three antibiotic resistance genes: *bla*_CTX-M_, *fosA*, and *tet*(34). Notably, the *bla*_CTX-M_ gene (8), intrinsic to *Kluyvera*, showed 91.03% amino acid identity to CTX-M-283. *In vitro* susceptibility testing was performed with the Vitek II system (bioMérieux, Marcy-l'Étoile, France) as per the manufacturer’s instructions. Results were interpreted per 2025 Clinical and Laboratory Standards Institute guidelines (9). Strain 142937 showed resistance to piperacillin, cefazolin, and cefuroxime, but was susceptible to amoxicillin/clavulanate, ticarcillin/clavulanate, ampicillin/sulbactam, piperacillin/tazobactam, ceftriaxone, cefepime, cefotaxime, ceftazidime, cefpodoxime, ertapenem, imipenem, meropenem, doripenem, amikacin, tobramycin, gentamicin, ciprofloxacin, levofloxacin, moxifloxacin, doxycycline, minocycline, tigecycline, and trimethoprim-sulfamethoxazole.

**Fig 1:**
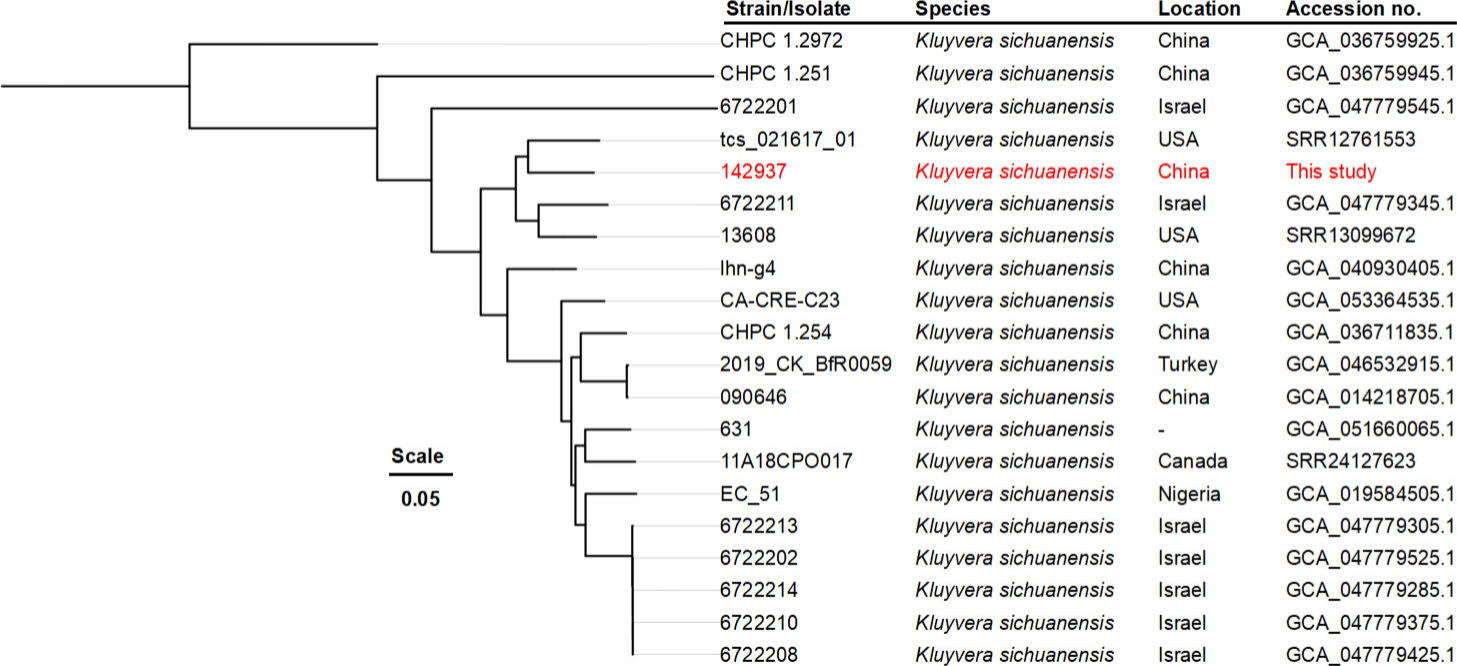
Phylogenomic tree of *K. sichuanensis*. Strain 142937 is labeled in red. Tree inferred from concatenated SNP alignments by IQ-Tree v2.4.0 under GTR model with gamma distribution and 1,000 bootstraps.

To explore clonal relationships between strain 142937 and other *K. sichuanensis*, we retrieved all available genomes from NCBI (*n* = 19, accessed on 11 November 2025). Using Snippy v4.6.0 (https://github.com/tseemann/snippy), we aligned them to the complete chromosome of *K. sichuanensis* strain lhn-g4 (CP162271). We then built a phylogenomic tree from concatenated single-nucleotide polymorphism alignments with IQ-Tree v2.4.0 (10), using GTR model with gamma distribution and 1,000 bootstraps. Software used default parameters. Phylogenetic analysis showed that strain 142937 clustered with three other *K. sichuanensis*: tcs_021617_01 (USA), 6722211 (Israel), and 13608 (USA). Notably, it differs from its closest relative (tcs_021617_01, SRR12761553) by 60,480 SNPs (Fig. 1), indicating significant divergence from other genome-available *K. sichuanensis* strains.

## Data Availability

The whole-genome sequence of 142937 was deposited in GenBank under the accession number JBTAOX000000000 and BioSample accession number SAMN53055789. The Illumina sequence reads were deposited in the Sequence Read Archive (SRA) database under accession number SRR35926721.
